# Supplementary material for: Manipulation of RNA polymerase III by Herpes Simplex Virus-1
Source: Nat Commun. 2022 Feb 2;13:623. doi: 10.1038/s41467-022-28144-8 (PMC8810925; doi:10.1038/s41467-022-28144-8)
Supplement: Supplementary file 1 — Supplementary Information [file 41467_2022_28144_MOESM1_ESM.pdf]

Manipulation of RNA Polymerase III by Herpes Simplex Virus-1

Sarah E. Dremel, Frances L. Sivrich, Jessica M. Tucker, Britt A. Glaunsinger, and Neal A. DeLuca

Supplementary Information

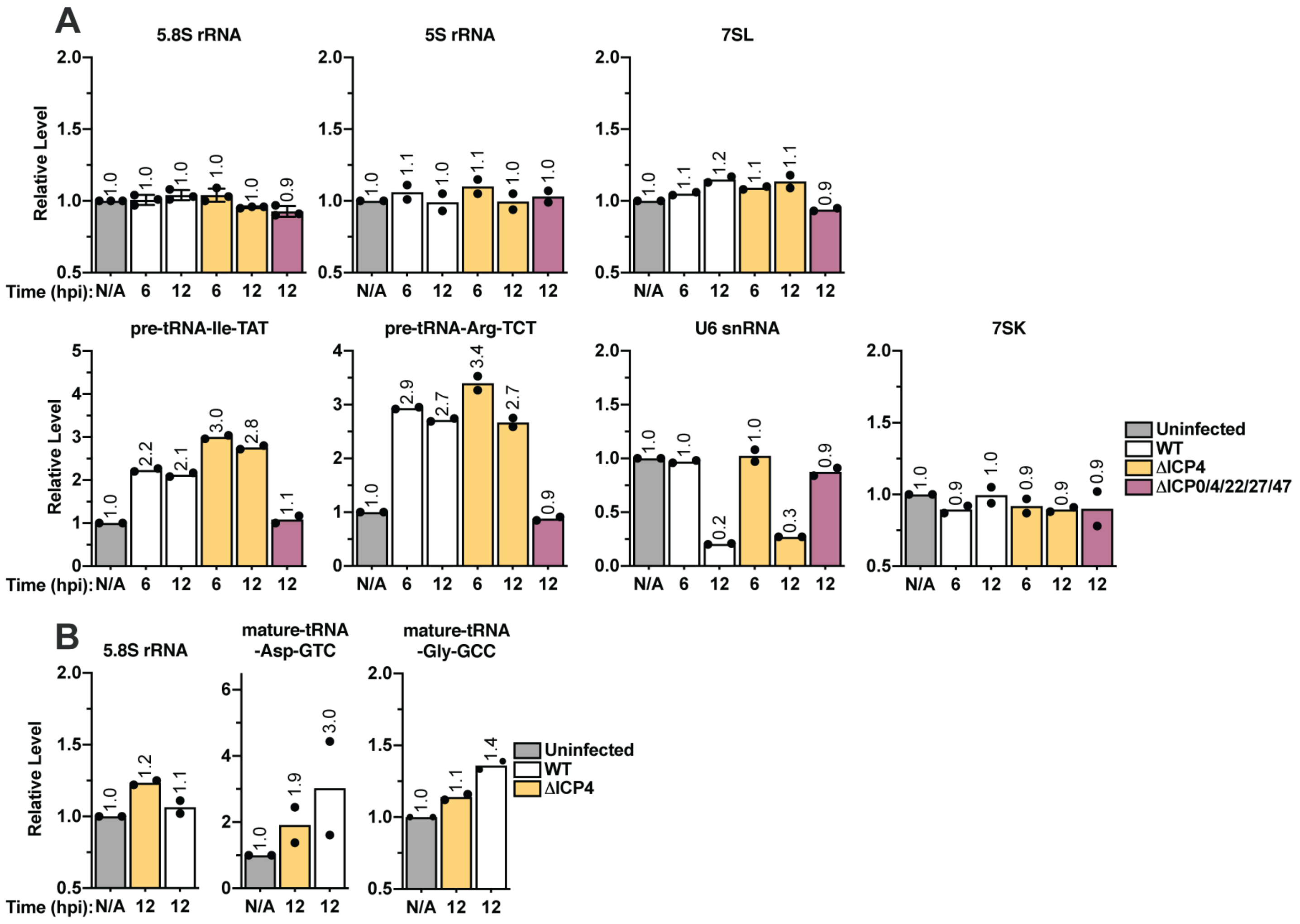

Supplementary Fig. 1 Quantified Northern blots for RNA Pol I and III transcripts after HSV-1 infection.

Human fibroblast cells were mock-infected or infected with ΔICP0/4/22/27/47 (d109), ΔICP4 (n12), or wild-type HSV-1 (KOS). RNA was isolated at indicated times, and Northern blots were used to assess transcript abundance. Data is plotted as "Relative Level", which is the signal from samples of interest relative to a matched uninfected sample. Data bars are the average, error bars are standard deviation, and each data point represents a biological replicate measurement. **a** Quantified Northern blot images for Fig. 1B (n=2 or 3). **b** Quantified Northern blot images for Fig. 1C (n=2).

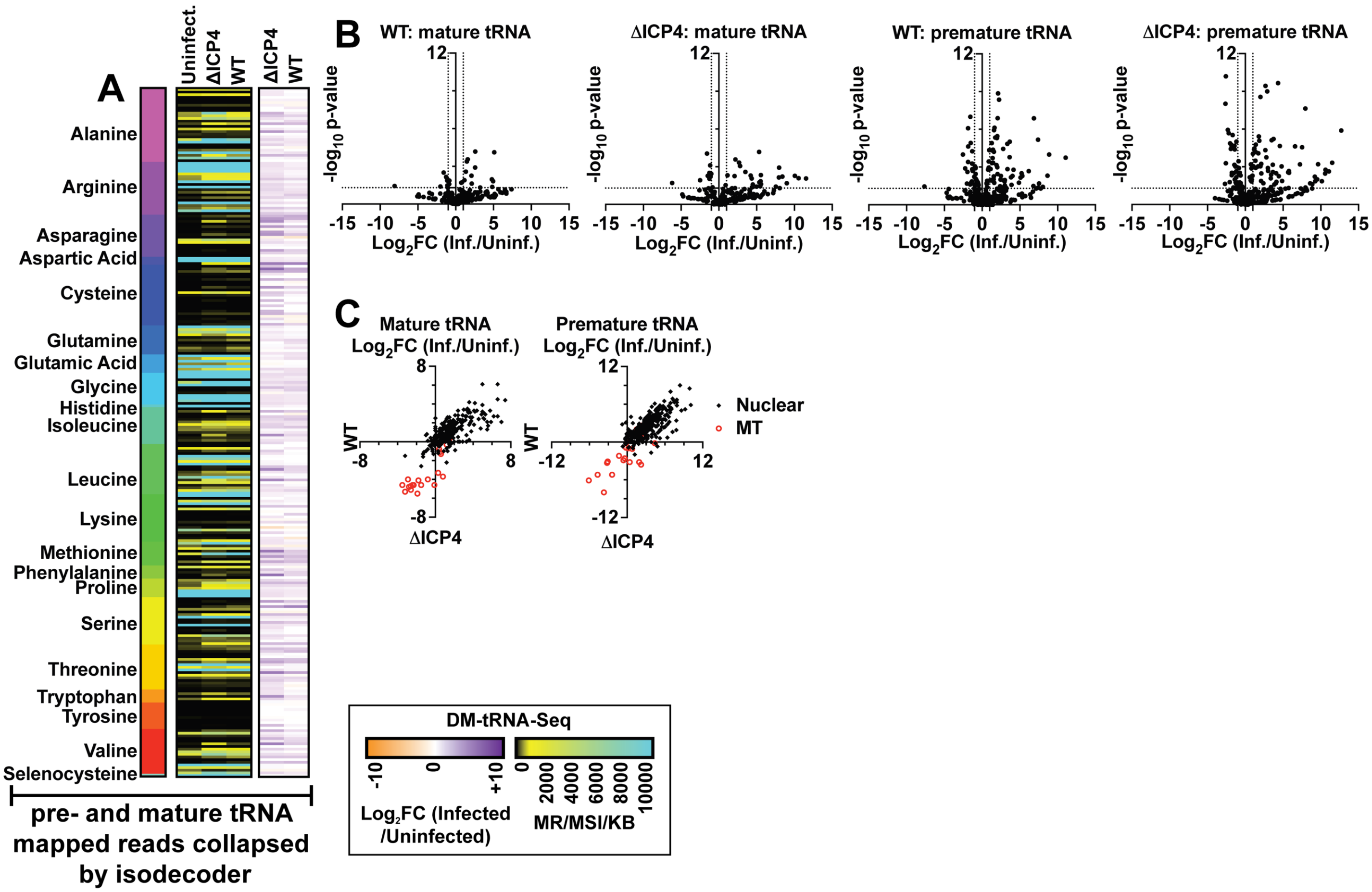

Supplementary Fig. 2 Comparison of tRNA isodecoder changes between WT and ∆ICP4 HSV-1 infection.

Human fibroblasts were mock-infected or infected with ∆ICP4 (n12) or wildtype HSV-1 and RNA was isolated at 12 hpi. DM-tRNA-Seq data was normalized to an internal spike-in control and the size in kilobase pairs (KB) of each tRNA (mapped reads per million spike-in reads per KB, MR/MSI/KB). We performed differential expression analysis (Benjamini-Hochberg) to calculate $\log_2$ fold change (infected/uninfected) and the false-discovery rate (FDR) p-value (n=4). **a** Comparison of tRNA isodecoder species upregulated during ∆ICP4 and wildtype HSV-1 infection. As tRNA genes are degenerate we summed pre- and mature-tRNA reads by target and anticodon (isodecoder). **b** Volcano plots of differentially expressed pre- and mature-tRNA species for ∆ICP4 or wildtype HSV-1. **c** tRNA expression changes ($\log_2$ fold change (infected/uninfected)) were plotted for wildtype (x-axes) and ∆ICP4 (y-axes) infection.

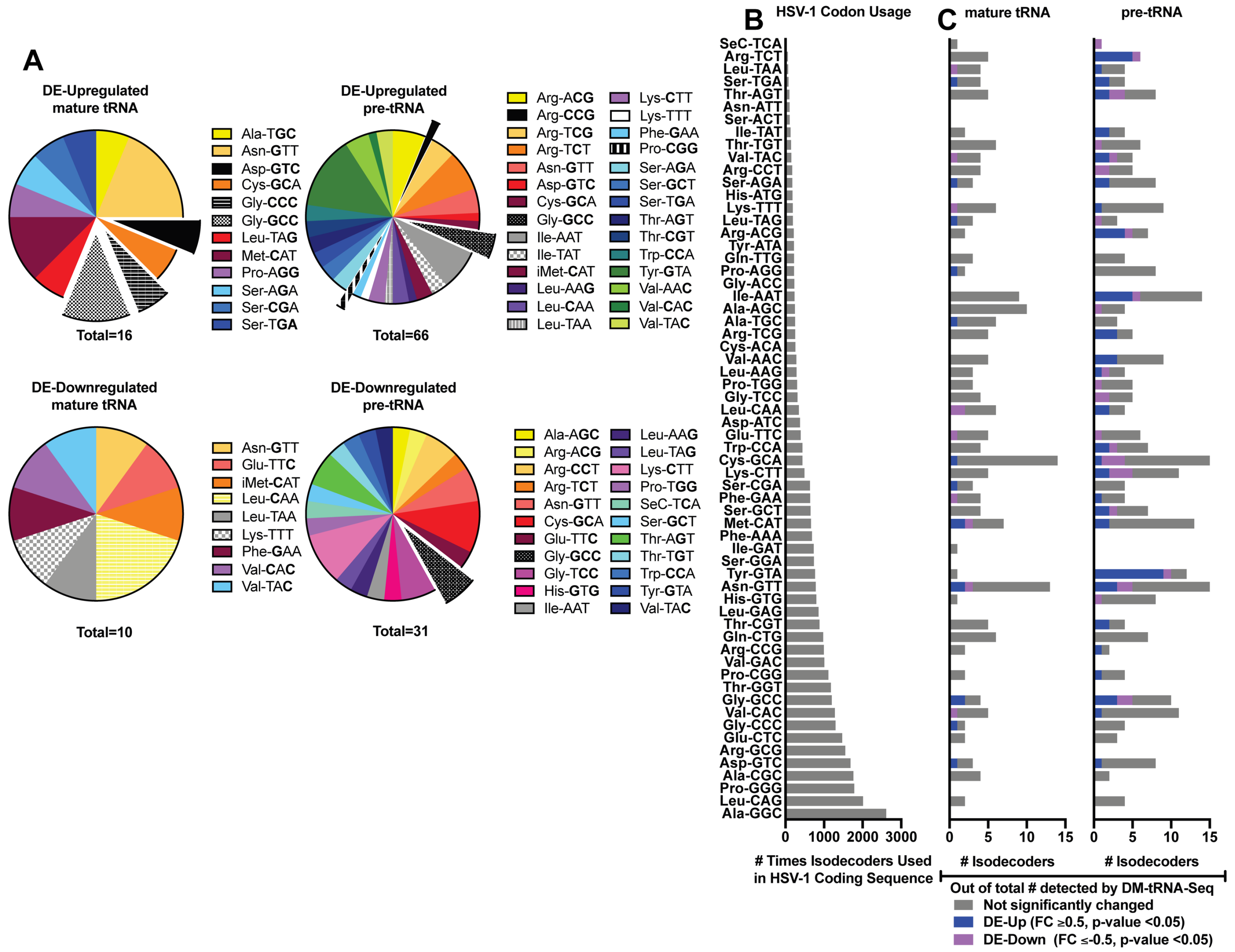

Supplementary Fig. 3 Distribution of tRNA isodecoders changed in HSV-1 infection. Analysis of isodecoder frequency among DE pre- and mature-tRNA from the DM-tRNA-Seq dataset. “DE-Up” had Log_2 fold change of wildtype HSV-1/uninfected of ≥0.5 and p-value of <0.05. “DE-Down” had Log_2 fold change of wildtype HSV-1/uninfected of ≤-0.5 and p-value of <0.05. As tRNA genes are degenerate we summarized by target and anticodon (isodecoder). **a** Distribution of affected tRNA isodecoders as a function of DE targets. Anticodons with a high GC content are exploded pieces. **b** Using the annotated coding sequence of HSV-1 we broke down the theoretical distribution of codon usage, assuming all viral proteins are made equally. Since the HSV-1 genome is ~68% GC content, isodecoder usage is skewed towards those with GC-rich codons. **c** Distribution of affected tRNA isodecoders as a function of those expressed in our experimental system. To classify a tRNA as expressed or “detected” in our experimental system, we required a cut off of at least 100 normalized mapped reads in uninfected or infected conditions.

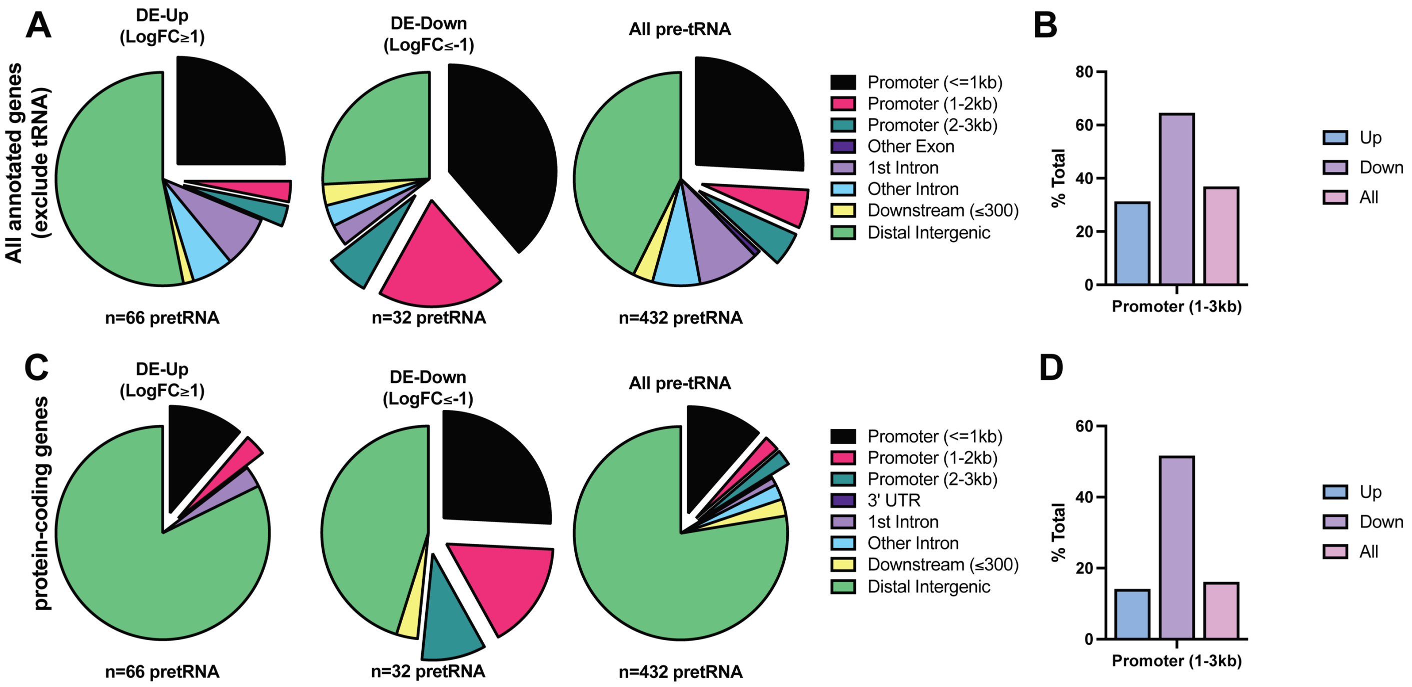

Supplemental Fig. 4 Relative localization of DE tRNA genes to other host genes.
Analysis of relative genomic position among DE pre- and mature-tRNA from the DM-tRNA-Seq dataset. "DE-Up" had Log_2 fold change of HSV-1/uninfected of ≥1 and p-value of <0.05. "DE-Down" had Log_2 fold change of HSV-1/uninfected of ≤-1 and p-value of <0.05. **a-b** Relative position was analyzed for DE tRNA to promoters from all annotated genes (hg38 gencode.v36), excluding tRNA. **c-d** Relative position was analyzed for DE tRNA to promoters from only protein-coding genes. **a, c** Relative position as a function of all tRNA analyzed. **b, d** Percentage of total targets that were located with 3 kb of a promoter.

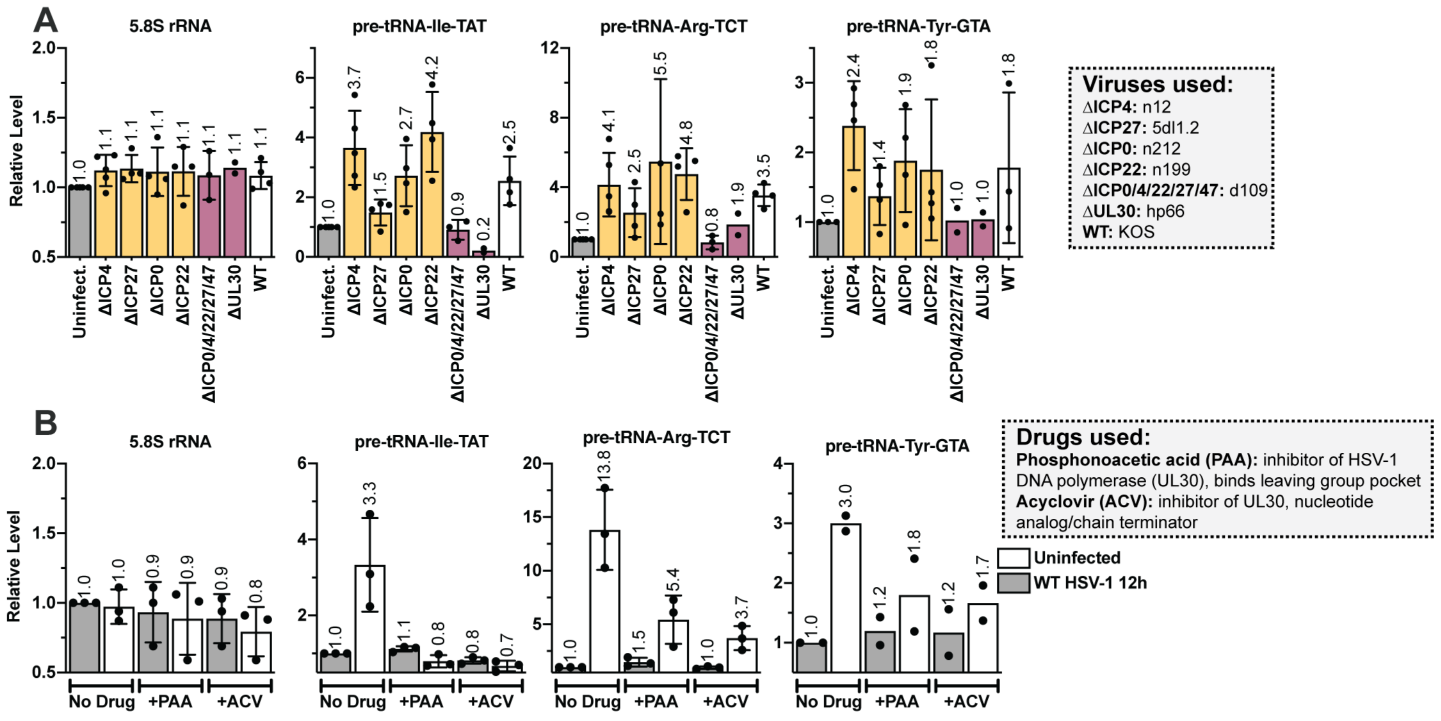

Supplementary Fig. 5 Determination of HSV-1 effectors and events required for tRNA upregulation.
Human fibroblast cells were mock-infected or infected with HSV-1 for 12 hours. RNA was isolated and Northern blots were used to assess transcript abundance. Blots are quantified as “Relative Level”, which is the signal from samples of interest relative to a matched uninfected sample. Data bars are the average, error bars are standard deviation, and each data point represents a biological replicate measurement. **a** Human fibroblasts were infected with wildtype HSV-1 (KOS) or a panel of HSV-1 mutants: ΔICP4 (n12), ΔICP27 (5dl1.2), ΔICP0 (n212), ΔICP22 (n199), ΔICP0/4/22/27/47 (d109), or ΔUL30 (hp66) (n=2-5). **b** At 0 hpi, 300 ug/mL phosphonoacetic acid (PAA) or 100 uM acyclovir (ACV) was used to treat mock-infected or cells infected with wildtype HSV-1 (KOS) (n=2 or 3).

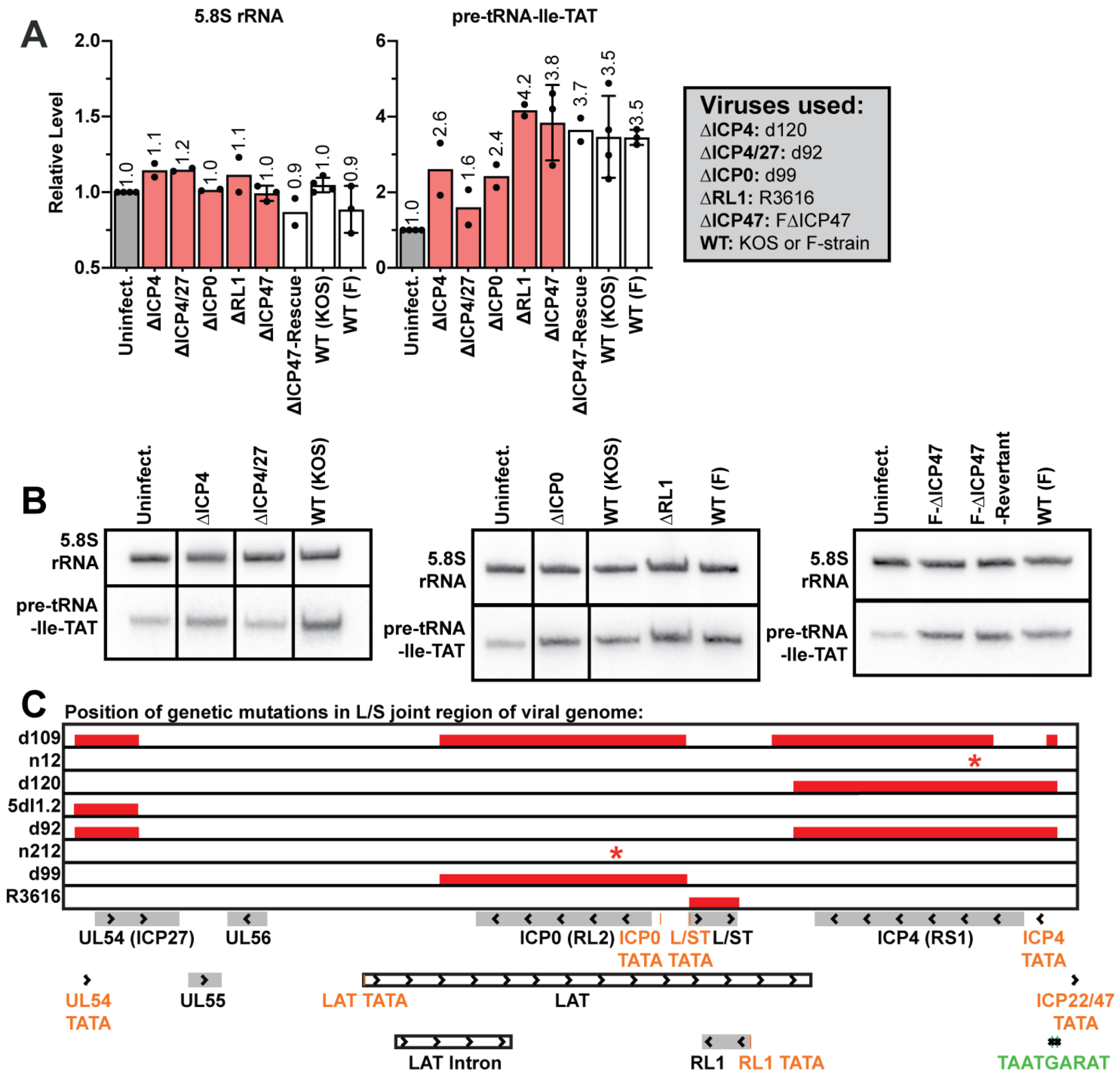

Supplementary Fig. 6 Requirement of differentially expressed HSV-1 genes in tRNA upregulation.

a-b Human fibroblast cells were mock-infected or infected with HSV-1 for 12 hours. RNA was isolated and Northern blots were used to assess transcript abundance. Fibroblasts with infected with wildtype HSV-1 (KOS, F-Strain, or FΔICP47-Revertant) or a panel of HSV-1 deletion mutants: ΔICP4 (d120), ΔICP4/27 (d92), ΔICP0 (d99), ΔRL1 (R3616), ΔICP47 (FΔICP47). **a** Blots are quantified as "Relative Level", which is the signal from samples of interest relative to a matched uninfected sample. Data bars are the average, error bars are standard deviation, and each data point represents a biological replicate measurement (n=2-4). **b** Representative Northern blot images from biological replicates. **c** Details of genetic mutants for viral mutants used in this study. Red boxes indicated deleted regions of the viral genome and red asterisks indicate the location of a nonsense point mutation inserted.

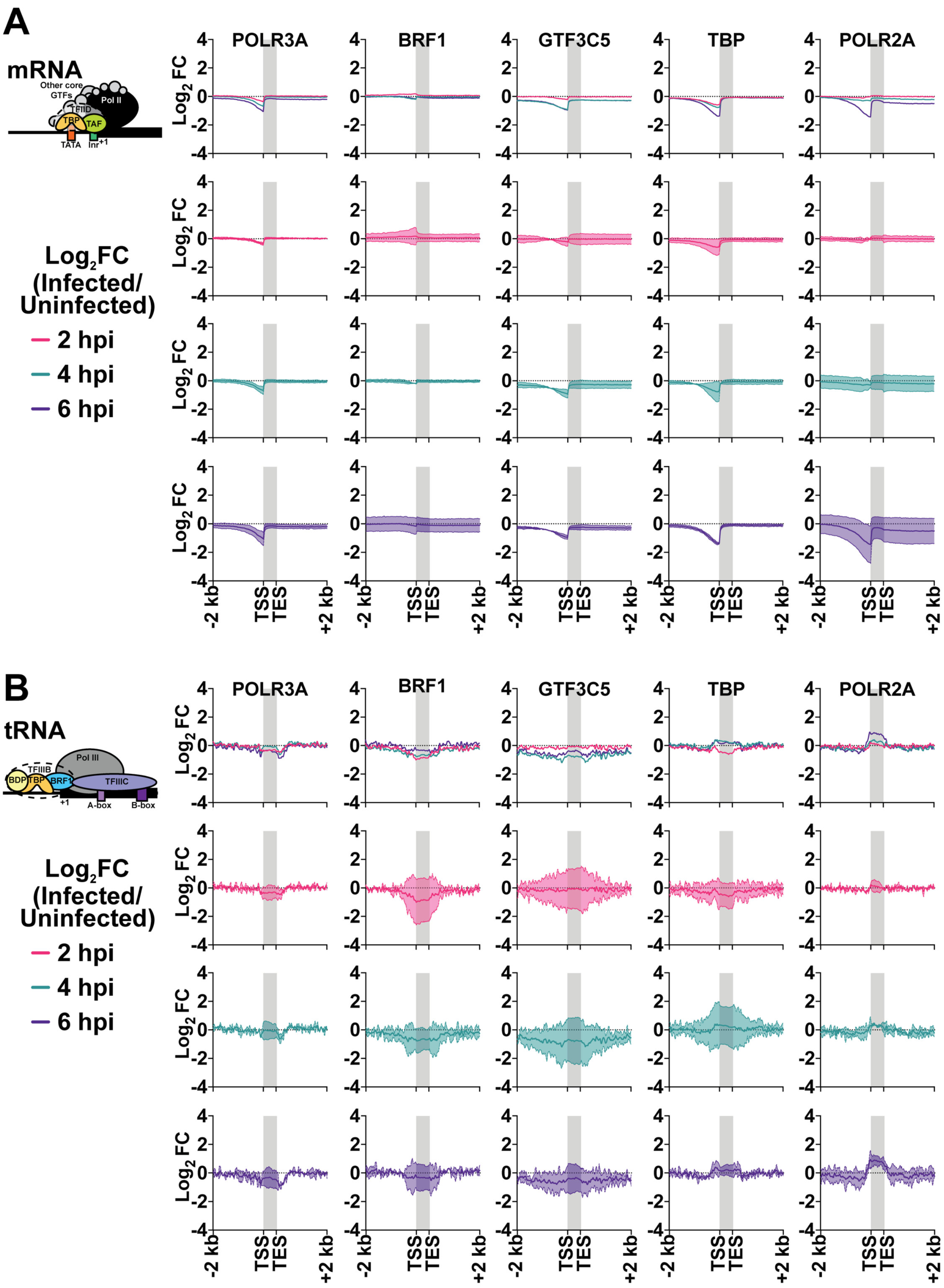

Supplementary Fig. 7 Analysis of reproducibility for Pol III GTF binding to host loci. Human fibroblasts were mock-infected or infected with HSV-1 for 2, 4, or 6 hours and ChIP-Seq was performed. ChIP-Seq data is normalized for sequencing depth and cellular genome sampling. Using normalized bigwigs we calculated $\log_2$ fold change against a paired uninfected sample for each biological replicate. We plotted the average $\log_2$ fold change from 2 kb upstream of TSS to 2 kb downstream of TES for all **a** mRNA (n=35608) or **b** tRNA loci (n=592). Data is the average of biological duplicates (solid line) with standard deviation (dashed line).

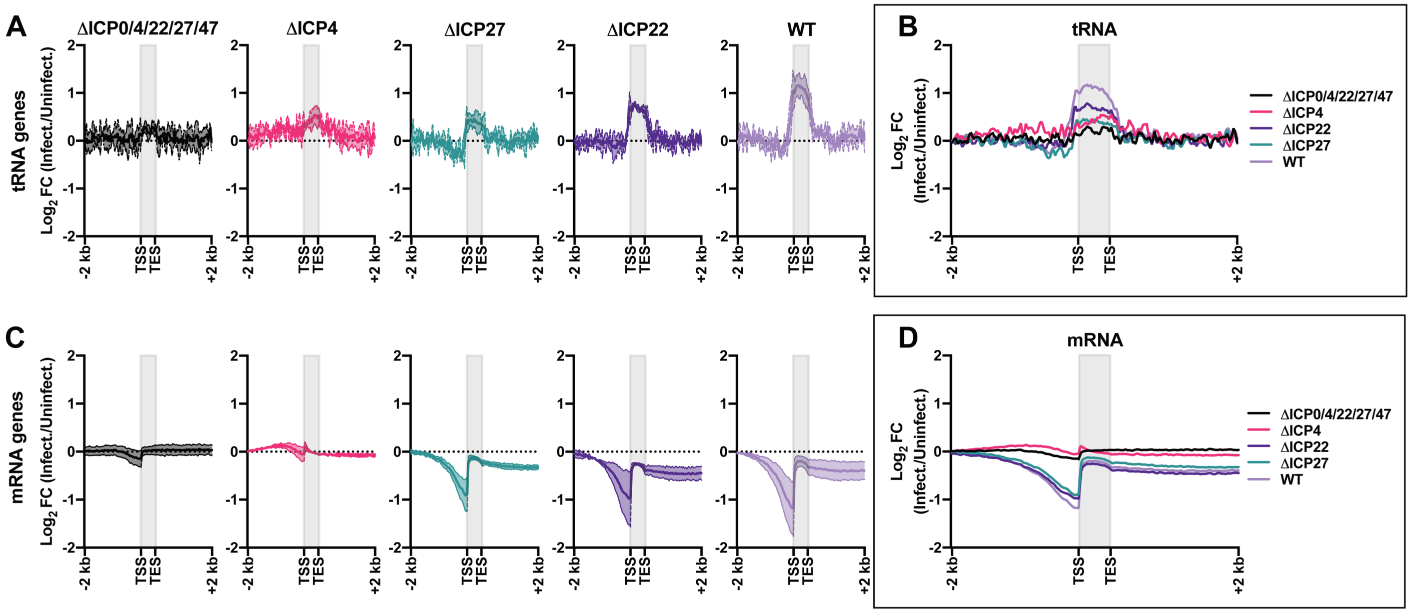

Supplementary Fig. 8 Pol II depletion from host mRNA and enrichment on tRNA are not linked. Human fibroblasts were mock-infected or infected with ΔICP0/4/22/27/47 (d109), ΔICP4 (n12), ΔICP27 (5dl1.2), ΔICP22 (n199), or wild-type HSV-1 and ChIP-Seq for POLR2A was performed. ChIP-Seq data is from biological triplicate experiments and normalized for sequencing depth and cellular genome sampling. Using normalized bigwigs we calculated $\log_2$ fold change against a paired uninfected sample for each biological replicate. We plotted the average $\log_2$ fold change from 2 kb upstream of TSS to 2 kb downstream of TES for all mRNA (n=35608) or tRNA loci (n=592). **a, c** Data plotted is average of biological triplicates (solid line) with standard deviation (dashed line). **b, d** Data plotted is average of biological triplicates.

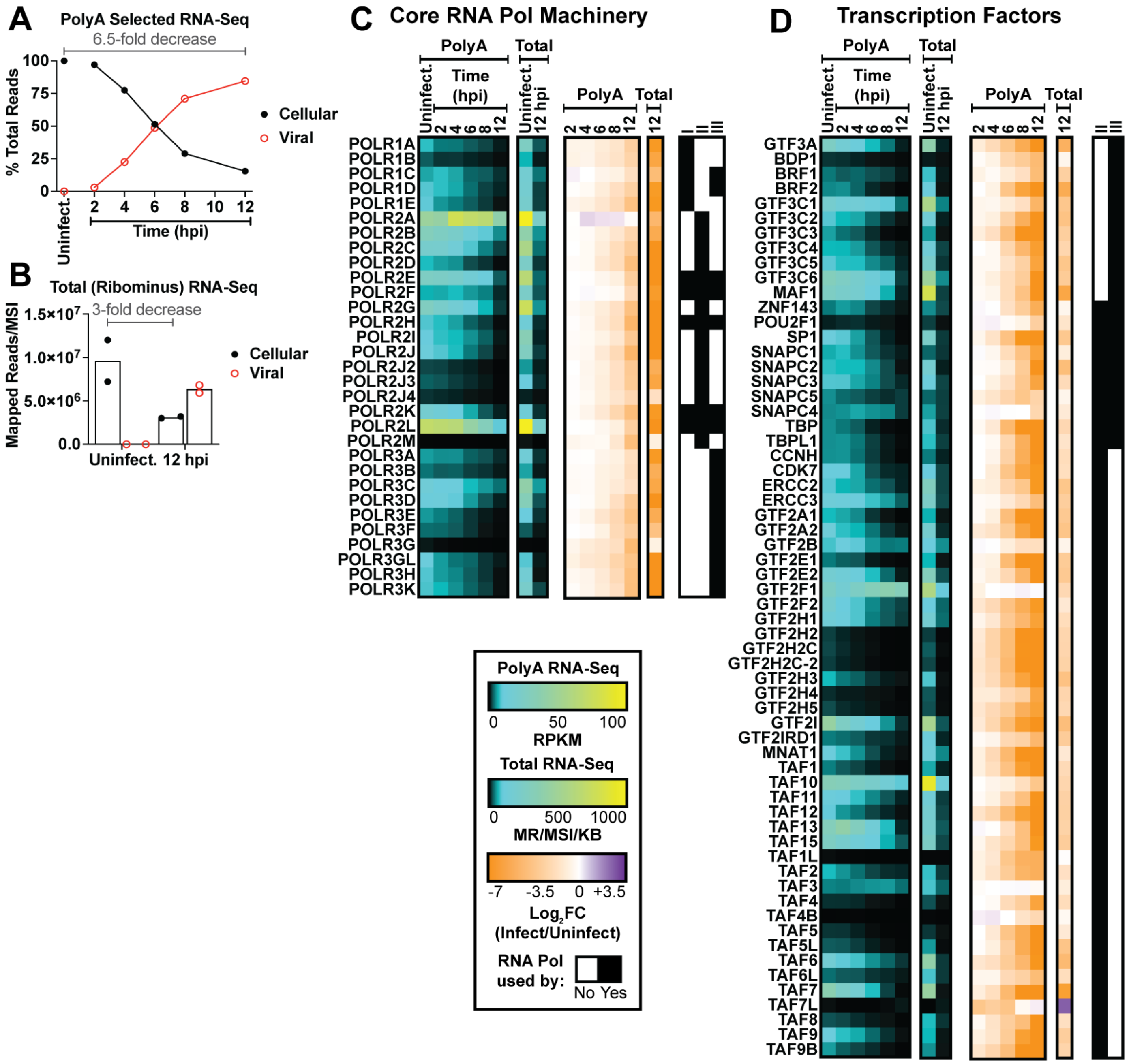

Supplementary Fig. 9 Transcriptomic changes of Pol II and III machinery during HSV-1 productive infection

Human fibroblasts were mock-infected or infected with wildtype HSV-1 for the indicated times. PolyA-selected or Total RNA-Seq was performed. Data is the average of biological duplicate experiments. **a** Global changes for polyA-RNA-Seq data are represented as reads mapped to viral or host transcripts per total reads sequenced (% Total Reads). Each data point is a biological replicate, columns are the average, and error bars are standard deviation (n=2). **b** Global changes for Total-RNA-Seq data are represented as reads mapped to viral or host transcripts per million spike-in reads. Each data point is a biological replicate, columns are the average, and error bars are standard deviation (n=2). **c-d** Heatmaps of transcript abundance for components of the Pol II and III transcriptional machinery. PolyA-RNA-Seq data is plotted as mapped reads per million total reads per kilobase pair (RPKM), or $\log_2$ fold change of infected over uninfected cells (Log_2FC). Total-RNA-Seq data is plotted as mapped reads per million spike-in reads per kilobase pair (MR/MSI/KB), or $\log_2$ fold change of infected over uninfected cells (Log_2FC).

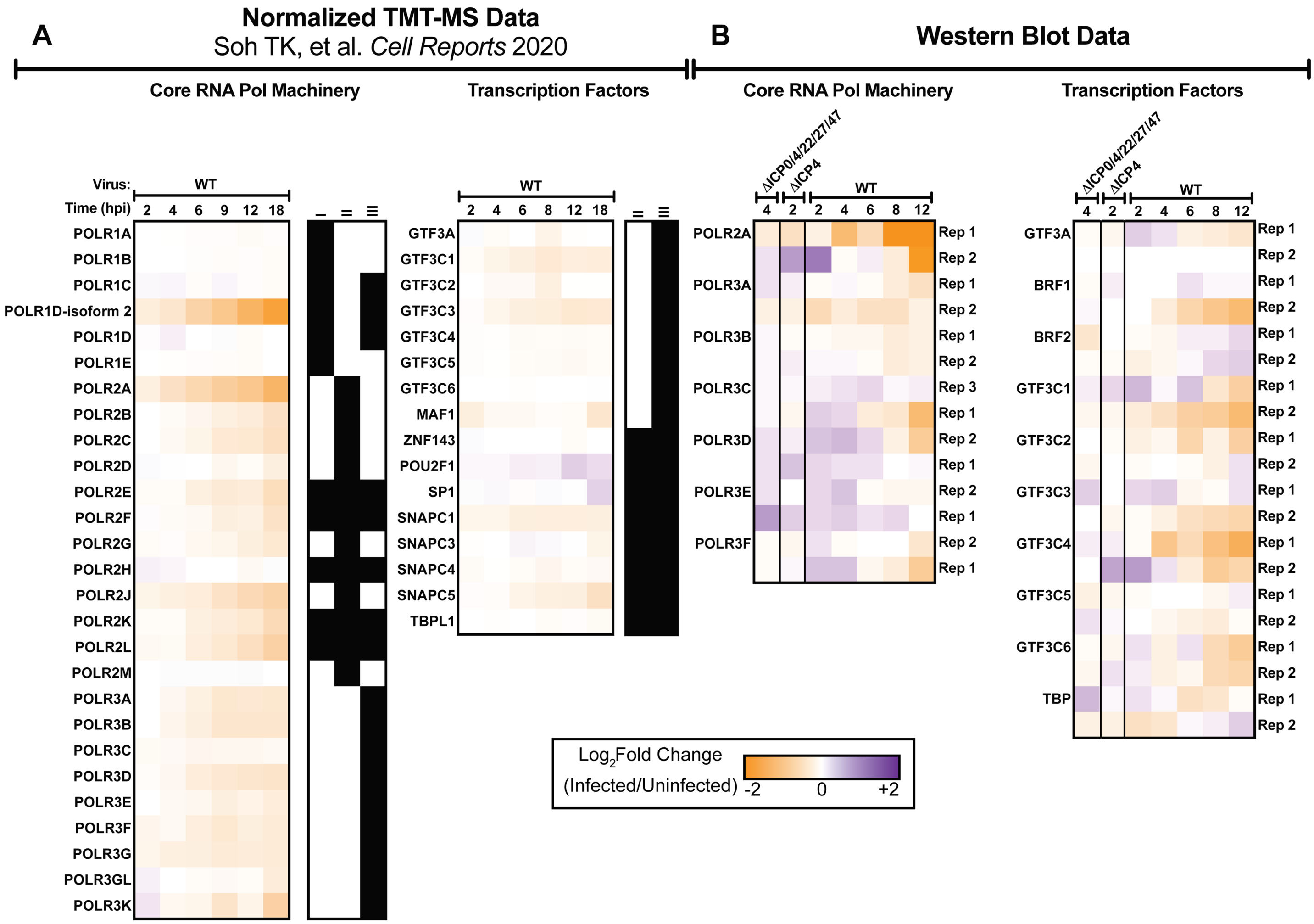

Supplementary Fig. 10 Proteomic changes of Pol II and III machinery during HSV-1 productive infection

a Tandem mass tag mass spectrometry (TMT-MS) data from mock- or HSV-1 infected HaCaT cells (human immortalized keratinocytes), published in[1]. **b** Human fibroblast (MRC5) cells were mock-infected or infected with ΔICP0/4/22/27/47 (d109) for 4 hours, ΔICP4 (n12) for 2 hours, or WT HSV-1 for 2, 4, 6, 8 or 12 hours. Protein was isolated and measured via LiCor-Western blot for indicated proteins. Each row is a biological replicate.

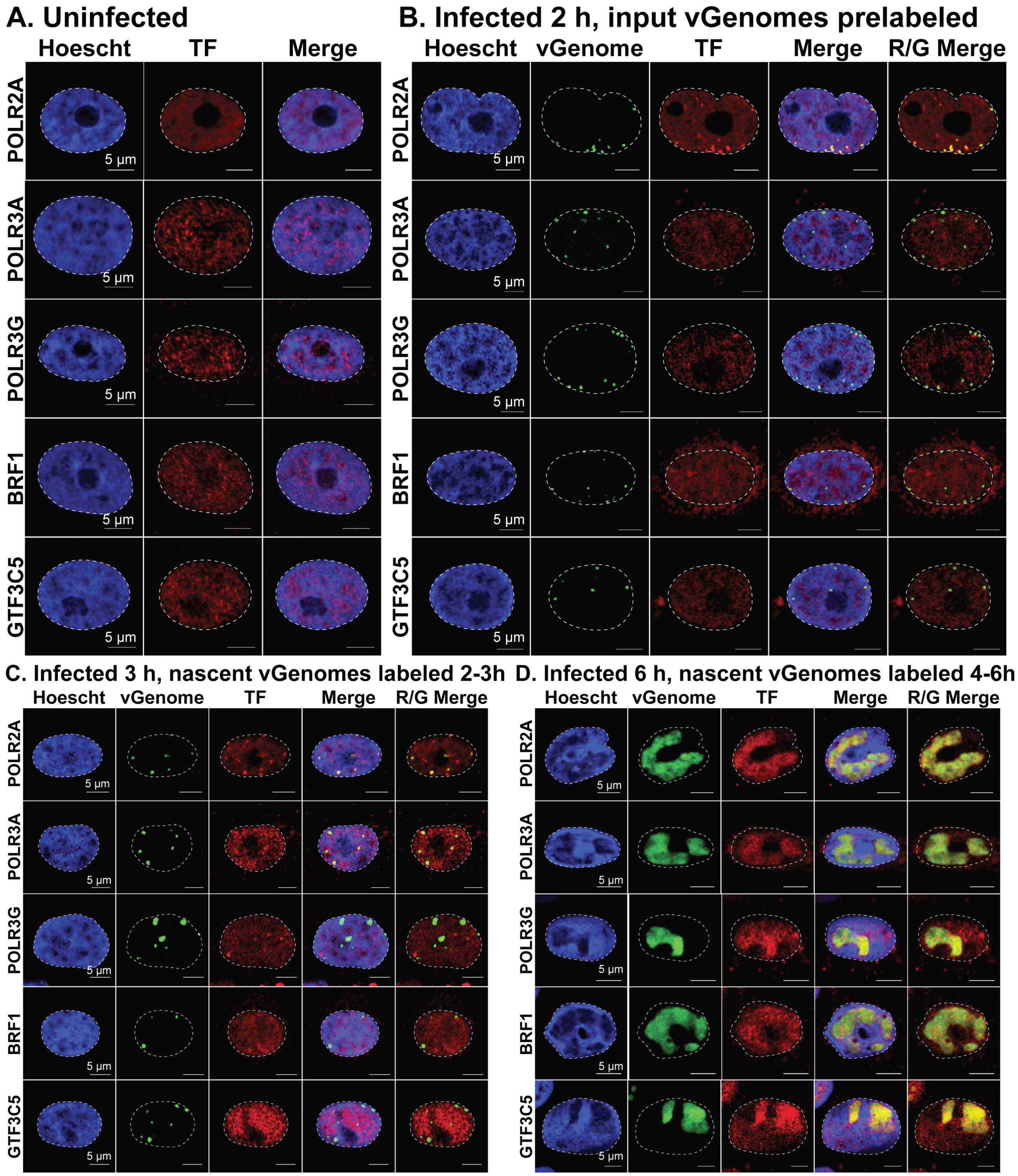

Supplementary Fig. 11 Altered nuclear organization of the Pol III transcription machinery
Vero cells were **a** mock-infected or **b-d** infected with HSV-1 for the indicated time. **b** To image input viral genomes we infected with an EdC-prelabed viral stock. **c-d** To image nascently replicated viral genomes we pulsed EdC at the indicated time points before fixing. Cells were fixed and EdC labeled DNA was tagged with alexa fluor 488 to visualize viral genomes (green) and cellular proteins were visualized by immunofluorescence (red). Nuclei were labeled with Hoechst (blue). Images are representative of biological duplicate experiments.

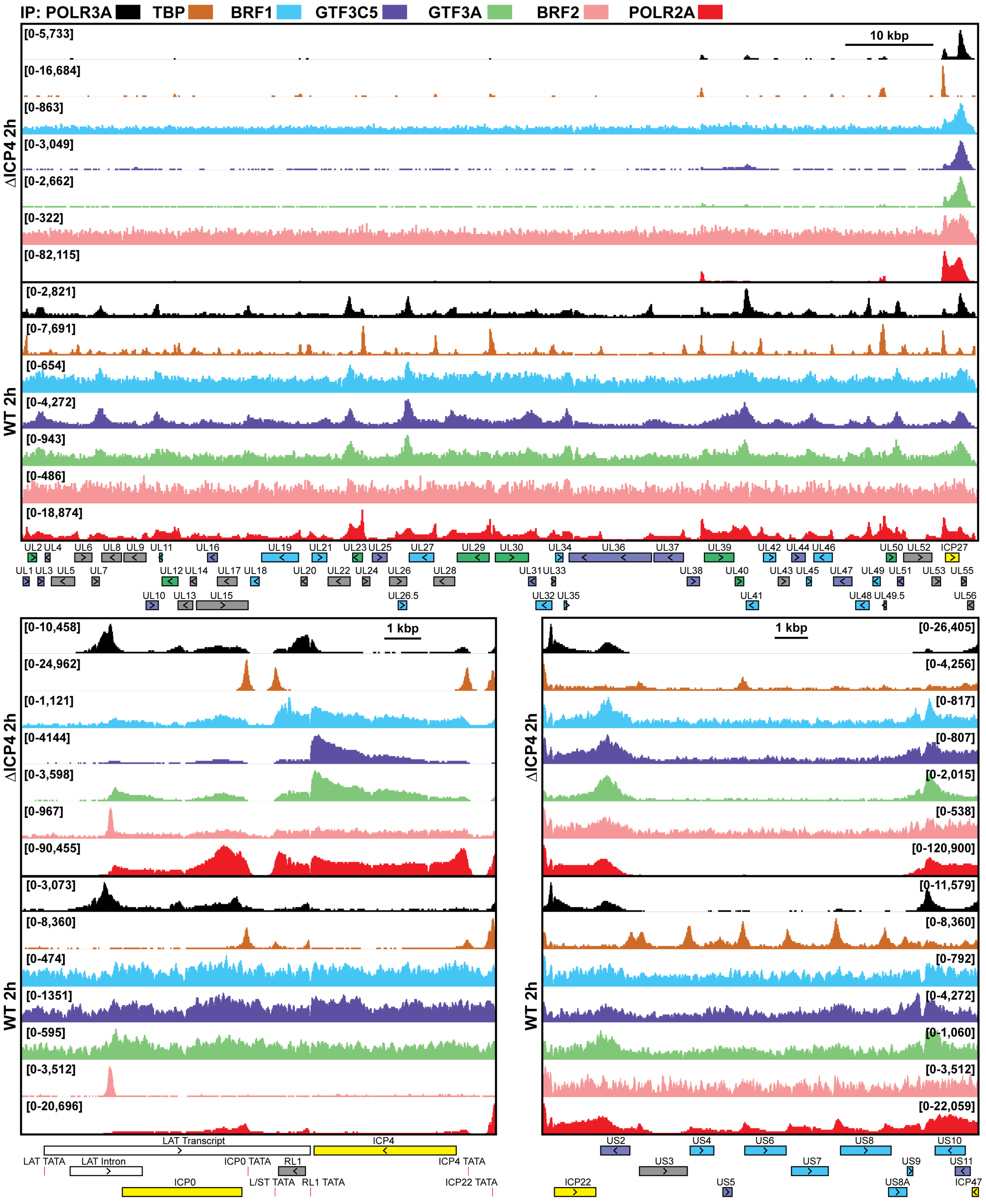

Supplementary Fig. 12 Pol III machinery recruitment to the HSV-1 genome during early infection

Human fibroblasts were infected with ∆ICP4 (n12) or wildtype HSV-1 for 2 h. ChIP-Seq was performed for POLR3A, POLR2A, TBP, GTF3A, GTF3C5, BRF1, and BRF2. Data is the average of biological duplicates and normalized for sequencing depth and viral genome copy number. Traces of binding to the unique long (UL), joint, and unique short (US) regions of the HSV-1 genome (KT899744.1 assembly). Y-axes maximum and minimum values are listed within brackets. Viral CDS are listed below, with colors indicating transcriptional class: immediate early (yellow), early (green), leaky late (blue), true late (purple), unclassified (grey). We have also annotated additional genomic features such as TATA boxes and ncRNA (white).

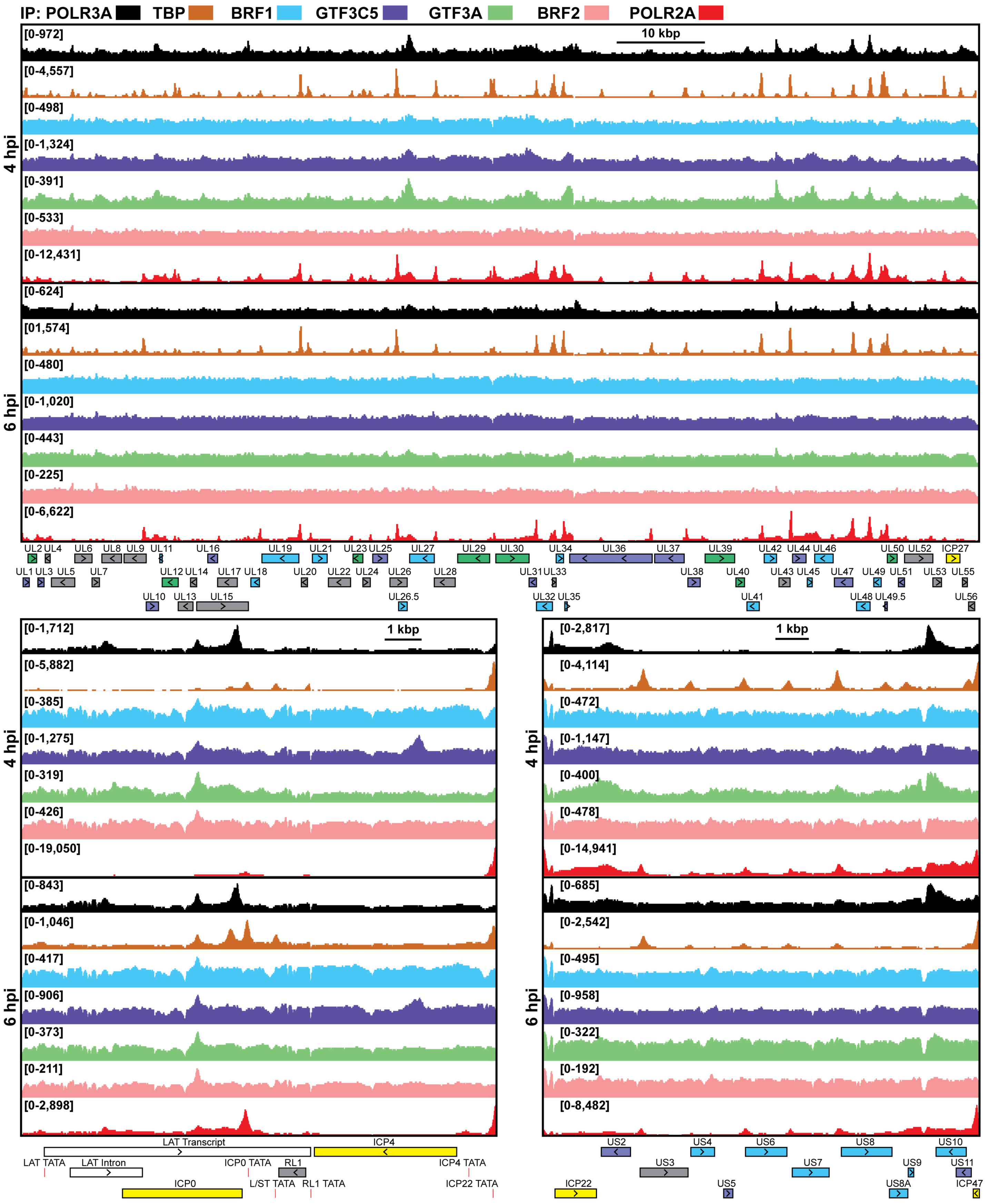

Supplementary Fig. 13 Pol III machinery recruitment to the HSV-1 genome post-viral genome replication

Human fibroblasts were infected with HSV-1 for 4 or 6h. ChIP-Seq was performed for POLR3A, POLR2A, TBP, GTF3A, GTF3C5, BRF1, and BRF2. Data is the average of biological duplicates and normalized for sequencing depth and viral genome copy number. Traces of binding to the unique long (UL), joint, and unique short (US) regions of the HSV-1 genome (KT899744.1 assembly). Y-axes maximum and minimum values are listed within brackets. Viral CDS are listed below, with colors indicating transcriptional class: immediate early (yellow), early (green), leaky late (blue), true late (purple), unclassified (grey). We have also annotated additional genomic features such as TATA boxes and ncRNA (white).

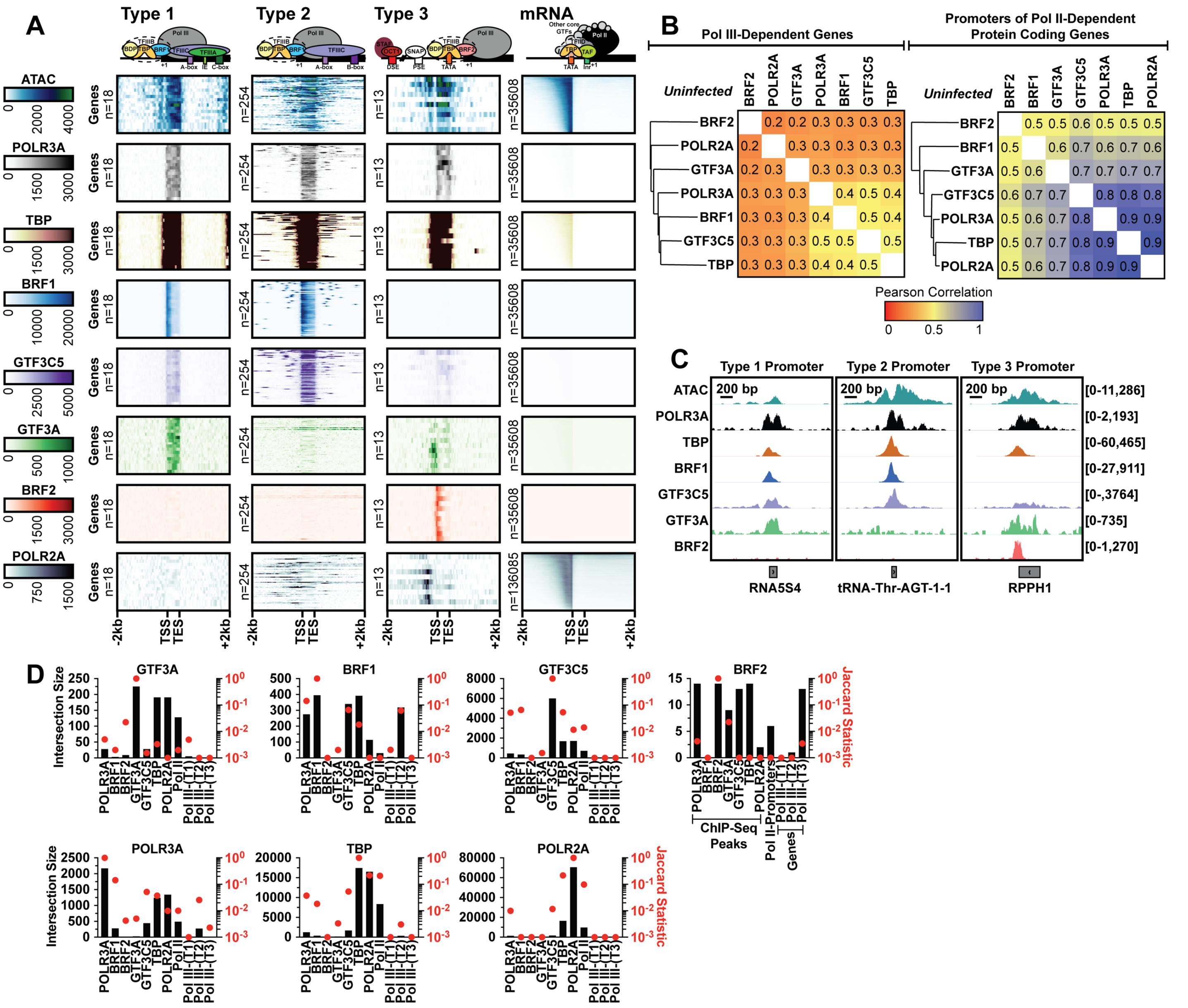

Supplementary Fig. 14 Recruitment of Pol III transcription machinery to the host genome. ATAC-Seq or ChIP-Seq for POLR3A, TBP, BRF1, GTF3C5, GTF3A, BRF2, and POLR2A was performed on uninfected human fibroblasts. Data is the average of biological duplicates and normalized to sequencing depth. **a** Heatmaps are from 2 kb upstream of TSS to 2 kb downstream of TES for loci, and "n" indicates the number of loci contained within each heatmap. Y-axes are mapped reads per billion total reads (MR/BTR). Models of canonical Pol III promoter architecture was adapted from[2]. **b** Pearson correlation analysis of binding profiles for the host genome. Analysis was limited to either Pol III-dependent loci (5S rRNA, tRNA, 7SL, 7SK, U6 snRNA, RMRP, BCYRN, RPPH1, SNAR, VT and Y transcripts) or 500 bp regions centered on protein-coding gene TSS. **c** Representative traces for factor binding at well characterized Type I, II, or III Pol III-dependent genes. Y-axes maximum and minimum values are listed within brackets. **d** Intersection analysis of host peak data relative to other factors tested and Type I, II, or III Pol III-dependent genes or 500 bp regions centered on protein-coding gene TSS.

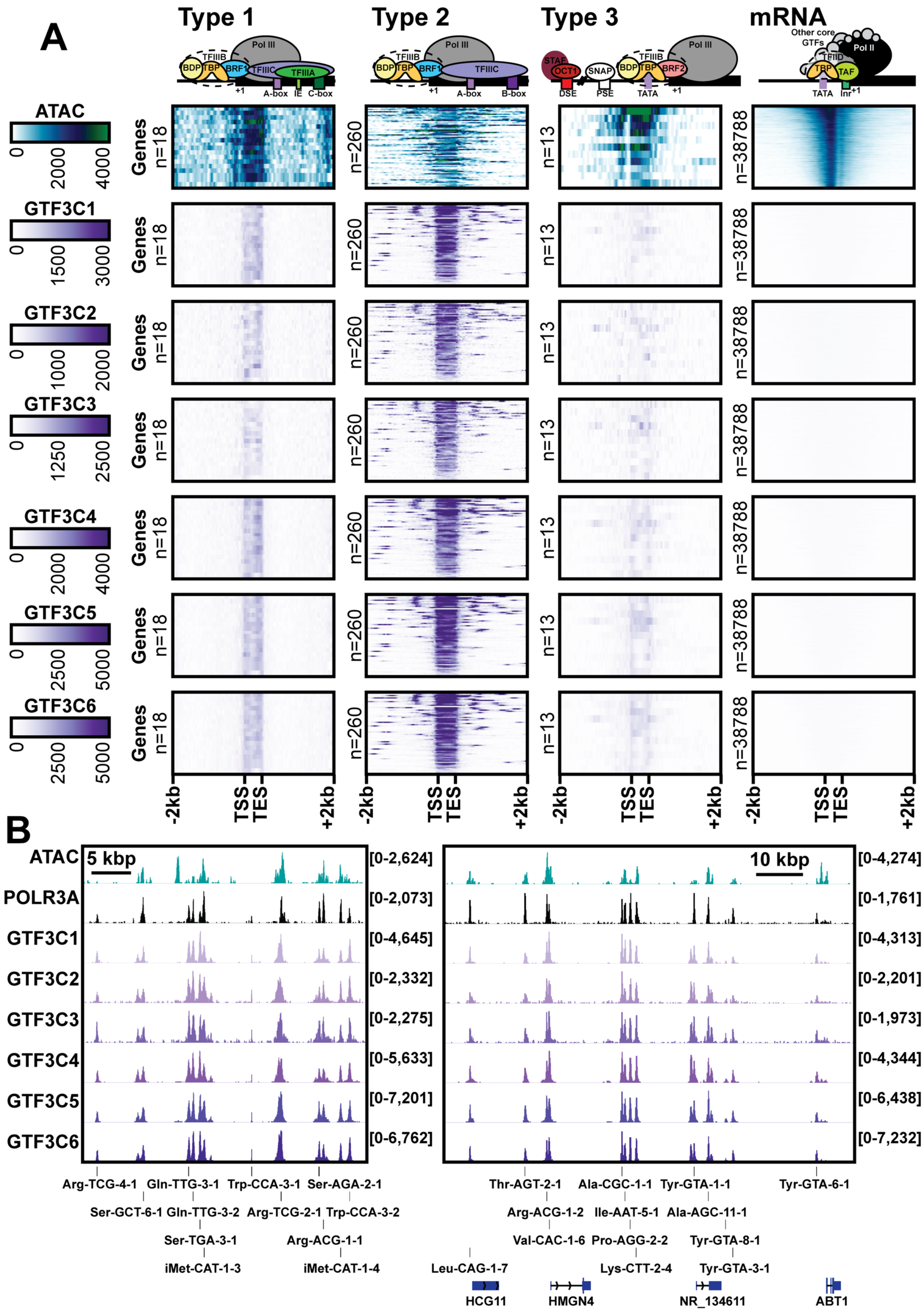

Supplementary Fig. 15 TFIIIC complex recruitment to the HSV-1 genome during early infection
Human fibroblasts were infected with HSV-1 for 2 h. ATAC-Seq or ChIP-Seq was performed for POLR3A, POLR2A, GTF3C1, GTF3C2, GTF3C3, GTF3C4, GTF3C5, and GTF3C6. Data is the average of biological duplicates and normalized for sequencing depth. **a** Heatmaps are from 2 kb upstream of TSS to 2 kb downstream of TES for loci, and "n" indicates the number of loci contained within each heatmap. Y-axes are mapped reads per billion total reads (MR/BTR). **b** Representative traces for factor binding at tRNA gene clusters located in chromosome 6. Y-axes maximum and minimum values are listed within brackets.

Supplementary Table 1. Antibodies Used in Study

Target	Antibody	Amount/Dilution Used		
		ChIP-Seq	Immunofluorescence	Western Blot
α-Tubulin	AbCam #ab7291	N/A	N/A	1:5000
Vinculin	Abcam #ab129002	N/A	N/A	1:15000
GAPDH	ThermoFisher #AM4300	N/A	N/A	1:5000
POLR2A	AbCam #ab5408	5 μg/IP	1:500	N/A
POLR2A	SantaCruz #sc899	N/A	N/A	1:500
TBP	AbCam #ab51841	25 μg/IP	N/A	1:1500
POLR3A	AbCam #ab96328	10 μg/IP	1:300	N/A
POLR3A	CST #12825S	N/A	N/A	1:500
POLR3B	AbCam #ab137030	N/A	N/A	1:250
POLR3C	Bethyl #A303-064A-M	N/A	N/A	1:250
POLR3D	AbCam #ab86786	N/A	N/A	1:500
POLR3E	Sigma #HPA041477	N/A	N/A	1:125
POLR3F	Abcam #ab180501	N/A	N/A	1:500
BRF1	SantaCruz #sc-390821	10 μg/IP	1:200	1:100
BRF2	SantaCruz #sc-390312	10 μg/IP	N/A	1:100
GTF3A	Abcam #ab254632	1 μg/IP	N/A	1:250
GTF3C1	Novus Biologicals #NB100-60657	10 μg/IP	N/A	1:500
GTF3C2	AbCam #ab89113	7.5 μg/IP	N/A	1:100
GTF3C3	SantaCruz #sc-101176	7.5 μg/IP	N/A	1:100
GTF3C4	Sigma #HPA069369	3 μg/IP	N/A	1:500
GTF3C5	Bethyl #A301-242A	3 μg/IP	1:500	1:500
GTF3C6	ThermoFisher #PA5-63948	7.5 μg/IP	N/A	1:100

Supplementary Table 2. Oligos Used in Study

Name	Sequence (5' to 3')	Used for
U6 snRNA	GAATTTGCGTGTCATCCTTGCGCAGGGGCCATGCTAA	Northern Blot
7SL	AACTTAGTGCGGACACCCGATC	Northern Blot
7SK	CGGGGAAGGTCGTCCTCTTC	Northern Blot
18S rRNA F	GTAACCCGTTGAACCCCATT	RT-qPCR
18S rRNA R	CCATCCAATCGGTAGTAGCG	RT-qPCR
45S pre-rRNA F	CTCCGTTATGGTAGCGCTGC	RT-qPCR
45S pre-rRNA R	GCGGAACCCTCGCTTCTC	RT-qPCR
Pre-tRNA Leu-CAA-2-1 F	ATGGCCGAGTGGTCTAAGG	RT-qPCR
Pre-tRNA Leu-CAA-2-1 R	ACCAGAAGACCCGAACACAG	RT-qPCR
Pre-tRNA Tyr-GTA-5-3 F	CCTTCGATAGCTCAGCTGGT	RT-qPCR
Pre-tRNA Tyr-GTA-5-3 R	CGACCTAAGGATGTCCACAAAT	RT-qPCR
Pre-tRNA Arg-TCT-2-1 F	GGCTCTGTGGCGCAATGGATA	RT-qPCR
Pre-tRNA Arg-TCT-2-1 R	TTCGAACCCACAACCTTTGAATTGCTC	RT-qPCR
tRNA iMet-CAT-1 F	CTGGGCCCATAACCCAGAG	RT-qPCR
tRNA iMet-CAT-1 R	TGGTAGCAGAGGATGGTTTC	RT-qPCR
5S rRNA F	GGCCATACCACCCTGAACGC	RT-qPCR
5S rRNA R	CAGCACCCGGTATTCCCAGG	RT-qPCR
7SL F	CAAAACTCCCGTGCTGATCA	RT-qPCR
7SL R	GGCTGGAGTGCAGTGGCTAT	RT-qPCR
U6 snRNA F	GGAATCTAGAACATATACTAAAATTGGAAC	RT-qPCR
U6 snRNA R	GGAACTCGAGTTTGCGTGTCATCCTTGCGC	RT-qPCR
tRNA Sec F	GGCTGATCCTCAGTGGTC	RT-qPCR
tRNA Sec R	GGTGGAATTGAACCACTC	RT-qPCR
7SK F	CGATCTGGTTGCGACATCTG	RT-qPCR
7SK R	CGTTCTCCTACAAATGGAC	RT-qPCR
BC200 F	ATAGCTTGAGCCCAGGAGTT	RT-qPCR
BC200 R	GCTTTGAGGGAAGTTACGCTTAT	RT-qPCR
PretRNA-Arg-TCT-2-1	TGCTCTATTCGTCACTAGAAGTCC	Northern Blot
Arg Unfolder	GCTATCCATTGCGCCACAGAGCC	Northern Blot
PretRNA-Ile-TAT-1-1	CCGCTCGCACTGTCATATAAGTACC	Northern Blot
Ile Unfolder	CTAACCGATTGCGCCACTGGAGC	Northern Blot
PretRNA-Tyr-GTA-5-1	GGATGTCTCCTGCTGAGGAAGTAGC	Northern Blot
Tyr Unfolder	TCCTTCGAGCCGGAATCGAACCAGCGACC	Northern Blot
Mature tRNA-Gly-GCC	TGGTGCATTGGCCGGGAATCGAACC	Northern Blot
Gly Unfolder	ATTCTACCACTGAACCACCAATGC	Northern Blot
Mature tRNA-Asp-GTC	TGGCTCCCCGTCGGGGAATCGAAC	Northern Blot
Asp Unfolder	CACTCACCACTATACTAACGAGGA	Northern Blot
5S	CGGTATTCCCAGGCGGTCT	Northern Blot
5.8S	CAATGTGTCCTGCAATTCAC	Northern Blot

Supplementary Table 3. Example of ChIP-Seq Normalization Method

Chromatin Set	IP	Sample	Total Mapped Reads (MR)	Host MR	HSV-1 MR	% Host	% HSV-1	Host Genome Copy #	HSV-1 Genome Copy #	Total Genome (Host+Viral) Content in Mbp	Host Norm Factor	HSV-1 Norm Factor
Rep1	Input	Uninfected	1.5E+07	1.5E+07	2.3E+03	100.0%	0.0%	2		6400	65	
Rep2			1.7E+07	1.7E+07	2.0E+02	100.0%	0.0%	2		6400	60	
Rep3			1.9E+07	1.9E+07	8.7E+02	100.0%	0.0%	2		6400	52	
Rep4			2.3E+07	2.3E+07	7.1E+02	100.0%	0.0%	2		6400	43	
Rep1		∆ICP4 2h	1.7E+07	1.7E+07	1.3E+05	99.2%	0.8%	2	319	6449	59	7.8
Rep2			2.1E+07	2.1E+07	8.7E+04	99.6%	0.4%	2	172	6426	47	11.5
Rep3			2.5E+07	2.5E+07	1.1E+05	99.5%	0.5%	2	194	6429	40	8.8
Rep4			2.4E+07	2.4E+07	1.0E+05	99.6%	0.4%	2	173	6426	41	10.0
Rep1		WT HSV-1 2h	1.5E+07	1.5E+07	5.2E+04	99.7%	0.3%	2	145	6422	66	19.3
Rep2			1.4E+07	1.4E+07	2.7E+04	99.8%	0.2%	2	83	6413	72	36.7
Rep3			2.4E+07	2.4E+07	7.7E+04	99.7%	0.3%	2	137	6421	42	13.1
Rep4			2.3E+07	2.3E+07	5.9E+04	99.7%	0.3%	2	106	6416	43	16.9
Rep1		WT HSV-1 4h	1.4E+07	1.3E+07	1.1E+06	92.3%	7.7%	2	3518	6935	78	0.9
Rep2			1.5E+07	1.5E+07	6.2E+05	96.0%	4.0%	2	1759	6667	68	1.6
Rep3			2.3E+07	2.2E+07	1.2E+06	94.7%	5.3%	2	2335	6755	45	0.8
Rep4			1.9E+07	1.9E+07	8.2E+05	95.7%	4.3%	2	1870	6684	54	1.2
Rep1		WT HSV-1 6h	1.7E+07	1.2E+07	4.9E+06	70.1%	29.9%	2	17991	9134	86	0.2
Rep2			1.6E+07	1.3E+07	3.2E+06	80.1%	19.9%	2	10444	7987	78	0.3
Rep3			2.5E+07	1.9E+07	6.1E+06	75.7%	24.3%	2	13484	8449	53	0.2
Rep4			2.1E+07	1.6E+07	4.8E+06	76.8%	23.2%	2	12714	8332	63	0.2

REFERENCES

1 Soh, T. K. *et al.* Temporal Proteomic Analysis of Herpes Simplex Virus 1 Infection Reveals Cell-Surface Remodeling via pUL56-Mediated GOPC Degradation. *Cell Reports* **33**, doi:10.1016/j.celrep.2020.108235 (2020).

2 Oler, A. J. *et al.* Human RNA polymerase III transcriptomes and relationships to Pol II promoter chromatin and enhancer-binding factors. *Nat. Struct. Biol* **17**, 620-629 (2010).
