# Supplementary material for: Manipulation of RNA polymerase III by Herpes Simplex Virus-1
Source: Nat Commun. 2022 Feb 2;13:623. doi: 10.1038/s41467-022-28144-8 (PMC8810925; doi:10.1038/s41467-022-28144-8)
Supplement: Supplementary file 3 — Description of Additional Supplementary Information [file 41467_2022_28144_MOESM3_ESM.pdf]

**Supplementary Data 1: DM-tRNA-Seq data.**

Human fibroblasts were mock-infected or infected with ∆ICP4 (n12) or wildtype HSV-1 for 12 hours. DM-tRNA-Seq was performed, data is the average of four data points consisting of two biological replicate experiments each containing two technical replicates. Data was normalized to an internal spike-in control and the size in kb of each tRNA.

**Supplementary Data 2: Transcription factor peaks on the viral genome.**

ChIP-Seq data was mapped to the HSV-1 genome (NCBI reference KT899744.1 with only one copy of repeat regions: ∆1-9063, 125845-126977, 145361-151974). Peaks were called using MACS2 and filtered by strength. For all factors except POLR2A, peak summits were extended 50 bp up- and down-stream from the summit. For POLR2A, the MACS2 narrow peaks output was recorded. Peaks are reported in bed3 format: chromosome, start, end.

**Supplementary Data 3: Transcription factor peaks on the host genome**

ChIP-Seq data was mapped to the human genome (NCBI reference hg38). Peaks were called using MACS2 with an FDR cutoff of 1% and the narrow peaks output was recorded.
