# Supplementary material for: Manipulation of RNA polymerase III by Herpes Simplex Virus-1
Source: Nat Commun. 2022 Feb 2;13:623. doi: 10.1038/s41467-022-28144-8 (PMC8810925; doi:10.1038/s41467-022-28144-8)
Supplement: Supplementary file 7 — Reporting Summary [file 41467_2022_28144_MOESM7_ESM.pdf]

nature portfolio

Corresponding author(s): Neal DeLuca
Last updated by author(s): Dec 5, 2021

Reporting Summary

Nature Portfolio wishes to improve the reproducibility of the work that we publish. This form provides structure for consistency and transparency in reporting. For further information on Nature Portfolio policies, see our Editorial Policies and the Editorial Policy Checklist.

Statistics

For all statistical analyses, confirm that the following items are present in the figure legend, table legend, main text, or Methods section.

n/a	Confirmed	
☐	☒	The exact sample size (n) for each experimental group/condition, given as a discrete number and unit of measurement
☐	☒	A statement on whether measurements were taken from distinct samples or whether the same sample was measured repeatedly
☐	☒	The statistical test(s) used AND whether they are one- or two-sided<br>*Only common tests should be described solely by name; describe more complex techniques in the Methods section.*
☐	☒	A description of all covariates tested
☒	☐	A description of any assumptions or corrections, such as tests of normality and adjustment for multiple comparisons
☐	☒	A full description of the statistical parameters including central tendency (e.g. means) or other basic estimates (e.g. regression coefficient) AND variation (e.g. standard deviation) or associated estimates of uncertainty (e.g. confidence intervals)
☐	☒	For null hypothesis testing, the test statistic (e.g. F, t, r) with confidence intervals, effect sizes, degrees of freedom and P value noted<br>*Give P values as exact values whenever suitable.*
☒	☐	For Bayesian analysis, information on the choice of priors and Markov chain Monte Carlo settings
☒	☐	For hierarchical and complex designs, identification of the appropriate level for tests and full reporting of outcomes
☒	☐	Estimates of effect sizes (e.g. Cohen's d, Pearson's r), indicating how they were calculated

Our web collection on statistics for biologists contains articles on many of the points above.

Software and code

Policy information about availability of computer code

Data collection	No software was used.
Data analysis	tRAX.v1 (https://github.com/UCSC-LoweLab/tRAX)<br>Galaxy v.21.05.1.dev0: CutAdapt v.3.4; SAMtools v.2; DeepTools v.3.3.2; Bowtie2 v.2.4.2; EdgeR v.3.34; HISAT2 v.2.2.1; Salmonquant v.1.5.1; bedtools v.2.30, FIMO v.5.3.2, MACS2 v.2.1.1.

For manuscripts utilizing custom algorithms or software that are central to the research but not yet described in published literature, software must be made available to editors and reviewers. We strongly encourage code deposition in a community repository (e.g. GitHub). See the Nature Portfolio guidelines for submitting code & software for further information.

Data

Policy information about availability of data

All manuscripts must include a data availability statement. This statement should provide the following information, where applicable:
- Accession codes, unique identifiers, or web links for publicly available datasets
- A description of any restrictions on data availability
- For clinical datasets or third party data, please ensure that the statement adheres to our policy

The DM-tRNA-Seq data generated in this study have been deposited in the SRA database under BioProject accession: PRJNA692681 or SRA Accession: SRR13484735, SRR13484733, SRR13484734.
The 4SU-Seq data generated in this study have been deposited in the SRA database under BioProject accession: PRJNA692715 or SRA Accession: SRR13484741, SRR13484740, SRR13484739, SRR13484738, SRR13484737, SRR13484736.
The Ribominus RNA-Seq data generated in this study have been deposited in the SRA database under BioProject accession: PRJNA732091 or SRA Accession:

SRR14632002, SRR14632001, SRR14632000, SRR14631999, SRR14631998, SRR14631997, SRR14631996, SRR14631995.
The PolyA-Selected RNA-Seq data generated in this study have been deposited in the SRA database under BioProject accession: PRJNA732134 or SRA Accession: SRR14632889, SRR14632890, SRR14632888, SRR14632887, SRR14632886, SRR14632885.
The ATAC-Seq data generated in this study have been deposited in the SRA database under BioProject accession: PRJNA553559 or SRA Accession: SRR10176079, SRR9661209, SRR10176081, SRR9661207.
The ChIP-Seq data generated in this study have been deposited in the SRA database.
BioProject accession: PRJNA553563 or SRA Accession: SRR9661306, SRR9661305, SRR9661304, SRR9661303, SRR9661302, SRR9661301.
BioProject accession: PRJNA693164 or SRA Accession: SRR13484792, SRR13484791, SRR13484790, SRR13484789, SRR13484788, SRR13484787, SRR13484785, SRR13484784, SRR13484783, SRR13484786, SRR13484782, SRR13484781, SRR13484779, SRR13484777, SRR13484776, SRR13484775, SRR13484778, SRR13484774, SRR13484773, SRR13484772.
BioProject accession: PRJNA732084 or SRA Accession: SRR14632140, SRR14632139, SRR14632138, SRR14632137, SRR14632143, SRR14632142, SRR14632141.
BioProject accession: PRJNA732212 or SRA Accession: SRR14633311, SRR14633310, SRR14633309, SRR14633308, SRR14633307, SRR14633306, SRR14633305, SRR14633304, SRR14633303, SRR14633302, SRR14633301, SRR14633300.
BioProject accession: PRJNA508787 or SRA Accession: SRR8288199, SRR8288200, SRR8288191, SRR8288192.

References used for sequence alignment are as follows:
Homo sapiens (NCBI: GRCh38.p13; https://www.ncbi.nlm.nih.gov/assembly/GCF_000001405.39)
Human herpesvirus 1 isolate KOS, complete genome (NCBI: KT899744.1; https://www.ncbi.nlm.nih.gov/nuccore/952947517/)

Field-specific reporting

Please select the one below that is the best fit for your research. If you are not sure, read the appropriate sections before making your selection.

☒ Life sciences ☐ Behavioural & social sciences ☐ Ecological, evolutionary & environmental sciences

For a reference copy of the document with all sections, see nature.com/documents/nr-reporting-summary-flat.pdf

Life sciences study design

All studies must disclose on these points even when the disclosure is negative.

Sample size	Number of biological replicates is specified in the methods section and/or figure legends. Sample size calculation was not performed. Kinetic time courses were performed in many instances, providing additional support for all conclusions. Sample size of two or greater was determined to be sufficient using correlation analysis of individual replicates.
Data exclusions	No data was excluded
Replication	All experiment were repeated with at least two biological replicates, and/or confirmed using orthogonal methods to ensure reproducibility.
Randomization	Randomization was not performed in this study. All experiments were done in individual tissue culture dishes with paired controls to ensure validity. We performed no animal or clinical work in this study that would require randomization
Blinding	Blinding was not relevant to this study as all measures were quantifiable by standard cellular or biochemical assays.

Reporting for specific materials, systems and methods

We require information from authors about some types of materials, experimental systems and methods used in many studies. Here, indicate whether each material, system or method listed is relevant to your study. If you are not sure if a list item applies to your research, read the appropriate section before selecting a response.

Materials & experimental systems

n/a	Involved in the study	
☐	☒	Antibodies
☐	☒	Eukaryotic cell lines
☒	☐	Palaeontology and archaeology
☒	☐	Animals and other organisms
☒	☐	Human research participants
☒	☐	Clinical data
☒	☐	Dual use research of concern

Methods

n/a	Involved in the study	
☐	☒	ChIP-seq
☒	☐	Flow cytometry
☒	☐	MRI-based neuroimaging

Antibodies

Antibodies used	Alpha-Tubulin: AbCam #ab7291<br>Vinculin: Abcam #ab129002<br>GAPDH : ThermoFisher #AM4300

POLR2A: AbCam #ab5408
POLR2A: SantaCruz #sc899
TBP: AbCam #ab51841
POLR3A: AbCam #ab96328
POLR3A: CST #12825S
POLR3B: AbCam #ab137030
POLR3C: Bethyl #A303-064A-M
POLR3D: AbCam #ab86786
POLR3E: Sigma #HPA041477
POLR3F: Abcam #ab180501
BRF1: SantaCruz #sc-390821
BRF2: SantaCruz #sc-390312
GTF3A: Abcam #ab254632
GTF3C1: Novus Biologicals #NB100-60657
GTF3C2: AbCam #ab89113
GTF3C3: SantaCruz #sc-101176
GTF3C4: Sigma #HPA069369
GTF3C5: Bethyl #A301-242A
GTF3C6: ThermoFisher #PA5-63948
Goat anti-Mouse IgG Alexafluor 594: Abcam #A32742
Goat anti-Rabbit IgG Alexafluor 594: Abcam #A32740
IRDye Goat anti-Mouse 680: LI-COR #926-68070
IRDye Goat anti-Mouse 800: LI-COR #926-32210
IRDye Goat anti-Rabbit 680: LI-COR #926-68071
IRDye Goat anti-Rabbit 800: LI-COR #926-32211

Validation

No additional validation statements were provided by the manufacturers
Western blot verification was performed for all antibodies confirming expected size of product and absence of non-specific bands.
ChIP-Seq target-based validation of POLR3A (AbCam), GTF3C1/2/3/4/5/6, GTF3A, BRF1, and BRF2 was performed in this study and shown in the supplementary data.

Eukaryotic cell lines

Policy information about cell lines

Cell line source(s)

Vero (African green monkey kidney, ATCC #CCL-81), U2OS (human osteosarcoma, ATCC #HTB-96), and MRC5 (human fetal lung, ATCC #CCL-171) cells were obtained from and propagated as recommended by ATCC. Additional Vero-based complementing cell lines were used to prepare and titer mutant virus stocks, this includes: E5 (ICP4+, n12, d120-complementing), E11 (ICP4/ICP27+, 5dl1.2, d92-complementing), F06 (ICP4/ICP27/ICP0+, d109-complementing), POLB3 (hp66-complementing). E5, E11, and F06 were generated in our lab and POLB3 cells were a kind gift from Don Coen.

Authentication

Cell lines did not undergo additional authentication after being received from supplier.

Mycoplasma contamination

Cell lines tested negative for mycoplasma contamination.

Commonly misidentified lines
(See ICLAC register)

No commonly misidentified lines were used in this study.

ChIP-seq

Data deposition

☒ Confirm that both raw and final processed data have been deposited in a public database such as GEO.

☒ Confirm that you have deposited or provided access to graph files (e.g. BED files) for the called peaks.

Data access links
May remain private before publication.

Data is submitted and successfully processed:
-HSV-1 POL3 Machinery-ChIP-Seq SRA: PRJNA693164
-HSV-1 GTF3C Complex-ChIP-Seq SRA: PRJNA732084
-ChIP-Seq for POLR2A in HSV-1 infected human fibroblasts SRA: PRJNA732212
Data is publicly available and was previously published:
ICP4 ChIP-Seq of WT (KOS) HSV-1 Productive Infection in MRC5 cells SRA: PRJNA553563

Files in database submission

Raw fastq files were uploaded for all samples to the SRA database
Bed files for ChIP-Seq peak binding data is included as supplementary data files (.xlsx)

Genome browser session
(e.g. UCSC)

N/A

Methodology

Replicates	At least two biological replicates were sequenced for all IP experiments. Paired input samples were sequenced for all IP's.
Sequencing depth	Illumina HiSeq 2500 platform was used to generate 50 bp SE reads. Sequencing depth was 10-30 million reads per sample.
Antibodies	POLR2A: AbCam #ab5408<br>TBP: AbCam #ab51841<br>POLR3A: AbCam #ab96328<br>BRF1: SantaCruz #sc-390821<br>BRF2: SantaCruz #sc-390312<br>GTF3A: Abcam #ab254632<br>GTF3C1: Novus Biologicals #NB100-60657<br>GTF3C2: AbCam #ab89113<br>GTF3C3: SantaCruz #sc-101176<br>GTF3C4: Sigma #HPA069369<br>GTF3C5: Bethyl #A301-242A<br>GTF3C6: ThermoFisher #PA5-63948
Peak calling parameters	MACS2: FDR cut off 1% (host), FDR cut off 5% (HSV-1)
Data quality	To assess quality and reproducibility of data we assessed normalized bigwig files for each IP replicate. For cellular and viral alignments we ran Deeptools MultiBigwigSummary with a bin size of 10,000 and 50 bp, respectively. Raw bin counts were plotted and a linear regression analysis was performed.
Software	Bowtie2, Deeptools
